# Relationship between iodine knowledge and dietary iodine intake in pregnant and lactating women: a cross-sectional study

**DOI:** 10.1017/S1368980023000514

**Published:** 2023-07

**Authors:** Jiaoyang Nie, Yuming Zhu, Chenchen Wang, Qin Lin, Rishalaiti Tayier, Zhuoxuan Cai, Pinjiang Ma, Ling Zhang

**Affiliations:** 1 School of Public Health, Xinjiang Medical University, Urumqi, People’s Republic of China; 2 Center for Disease Control and Prevention of Xinjiang Uygur Autonomous Region, Urumqi 830002, People’s Republic of China

**Keywords:** Iodine status, Iodine knowledge, Dietary iodine intake, Pregnant and lactating women, Urinary iodine concentration

## Abstract

**Objective:**

This study assessed the iodine knowledge of pregnant and lactating women and the relationship to dietary iodine intake and iodine status. The factors influencing iodine intake were analysed.

**Design:**

Basic information and iodine knowledge were collected via a questionnaire. A FFQ assessed dietary iodine intake. The urinary iodine concentration (UIC) was measured using the arsenic-cerium catalytic spectrophotometric determination of iodine in urine (WS/T 107 -2016).

**Setting:**

A cross-sectional study involving pregnant and lactating women in Xinjiang, China was conducted.

**Participants:**

A total of 1181 pregnant women and 504 lactating women were enrolled in the study.

**Results:**

The median UIC for pregnant and lactating women was 179·27 and 192·81 µg/l, respectively, and the dietary iodine intake was 407·16 and 356·89 µg/d, respectively. Of the pregnant and lactating women, 73·4 % and 82·5 % had medium iodine knowledge, respectively. In pregnant women, iodine knowledge and dietary iodine intake were positively correlated. High iodine knowledge and iodine education were shown to be protective factors for excessive iodine intake in pregnant women.

**Conclusion:**

This study demonstrated that the iodine nutritional status of women in Xinjiang was appropriate, and iodine knowledge was at a medium level, but there was confusion about iodine nutrition. Public education is needed to improve iodine knowledge and active iodine supplementation awareness among these populations of women.

Iodine is a micronutrient necessary for the body to synthesise thyroid hormones (TH), which control and regulate metabolism. In addition, TH are essential for normal foetal growth in utero and neurologic growth and development of the newborn^(1–3)^. Dietary iodine is the main source of iodine for humans. Pregnant and lactating women are vulnerable to iodine deficiency and have a greater need for iodine than non-pregnant women. According to the WHO, the UNICEF and the Iodine Global Network, the recommended iodine intake for pregnant and lactating women is 250 µg/d^(4)^, which ensures that the mother has sufficient iodine to increase TH production, meet foetal needs and increase renal clearance^(5,6)^.

Iodine deficiency disorders (IDD) are described as preventable neurologic disorders^(7)^. According to the WHO, the median urinary iodine concentration (MUIC) is the best marker for assessing iodine nutrition in pregnant women because > 90 % of dietary iodine is excreted in the urine^(4,8)^. Dietary iodine intake is considered to be the most direct biomarker of iodine status in populations and individuals^(9,10)^. Inadequate iodine intake during pregnancy and lactation may lead to maternal and foetal hypothyroidism and may impair foetal neurodevelopment, while severe iodine deficiency may have more serious effects, such as cretinism^(2)^. Because iodine in the body of pregnant and lactating women is mainly consumed from the diet, nutritional knowledge and attitudes are important factors in dietary habits. It has been suggested that a lack of knowledge and information about iodine may be a risk factor for iodine deficiency in pregnant and lactating women^(10)^. In addition, low iodine knowledge and negative attitudes about iodine are associated with the risk of a low urinary iodine concentration (UIC)^(11–13)^.

The global strategy to correct IDD through salt iodisation programmes and food iodine fortification initiatives has yielded significant results. Since the implementation of universal salt iodisation in 1994, iodine nutrition in China has been greatly improved^(14)^. In the past two decades, IDD awareness among Chinese populations has steadily increased, with universal salt iodisation as the main focus, supplemented by health promotion and education^(15)^; however, the results achieved have not led to increased public knowledge about iodine nutrition^(10,16)^. Studies involving the relationship between iodine status and knowledge in pregnant and lactating women are limited in China. A search of the literature revealed one study on iodine intake and knowledge among pregnant women in Zhejiang Province^(17)^. Adequate knowledge about iodine nutrition by pregnant and lactating women helps women to proactively consume sufficient iodine from the diet to ensure that maternal and foetal iodine needs are met. Moreover, improving public awareness and understanding of iodine-related knowledge may significantly reduce IDD.

Xinjiang is a vast region, far from the sea, with a general iodine deficiency in the external environment and was once a heavily endemic area for IDD. The prevention of IDD has become more critical since the emergence of new cases of cretinism in the southern Xinjiang region in 2006. Due to differences in the natural environment, lifestyle, dietary habits and economic conditions in the different areas of Xinjiang, some people’s awareness of IDD prevention still needs to be improved^(18–20)^. Accordingly, the current study describes the iodine knowledge and status of pregnant and lactating women in Xinjiang. We also assessed the relationship between iodine knowledge scores and intake and determined the potential factors influencing dietary iodine intake in pregnant and lactating women.

## Materials and Methods

### Participants and study design

**Fig. 1:**
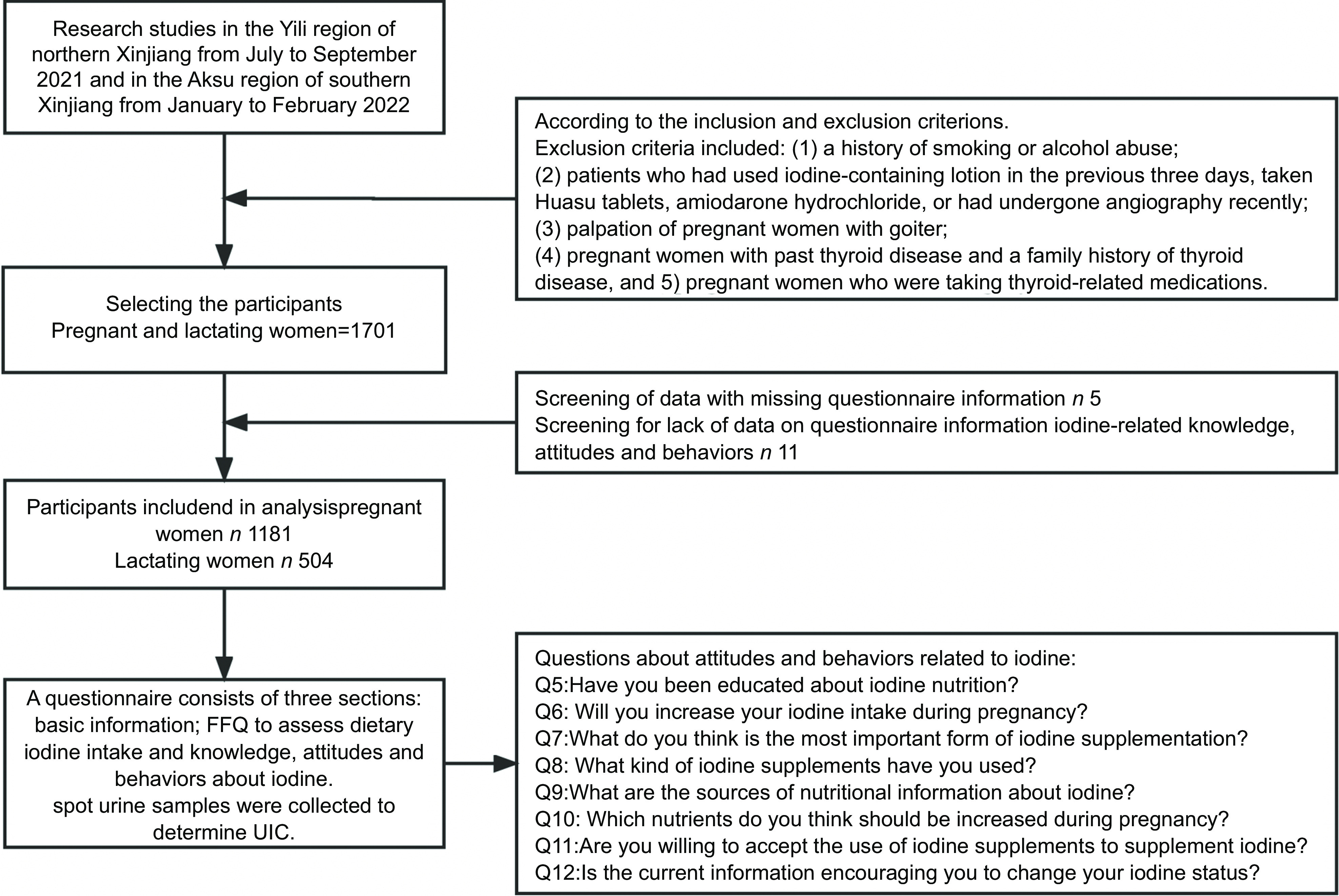
Participant flow chart. The figure shows a flow chart of the participants in this study and the specific questions (Q5–12) regarding attitudes and behaviours towards iodine

This cross-sectional study included pregnant and lactating women from two different geographic regions (southern and northern Xinjiang) in Xinjiang, China. The data collection was conducted from July to September 2021 in four counties in the Yili region of northern Xinjiang, and from January to February 2022 in three counties in the Aksu region of southern Xinjiang in each county (city and district) based on east, west, north, south and centre directions of each randomly selected township (town and street). Following the principle of informed and voluntary consent, and inclusion and exclusion criteria, fifty pregnant and twenty lactating women were selected from each township (town and street) to participate in the survey. The selected lactating women were those who were predominantly breastfeeding and had been breastfeeding for ≤ 1 year. (Fig. 1). The staff informed each participant of the study results.

### Questionnaire design and assessment

Data were obtained during face-to-face interviews using a questionnaire consisting of three sections. The first section focussed on the socio-demographic characteristics of the participants (age, BMI, education level, occupation, annual household income and iodised salt consumption), the existence of thyroid conditions and lifestyle habits during pregnancy and lactation (alcohol consumption and passive smoking status). The first section also included questions regarding the current pregnancy of the participants (expected date of delivery, parity and number of abortions).

The second section of the questionnaire assessed the dietary iodine intake of participants. Before the survey began, researchers collected several types of commercially available foods based on the dietary habits of residents in the Yili and Aksu regions of northern and southern Xinjiang, including the following eleven major food groups: staple foods; soybean products; livestock and poultry meat; eggs; dairy products; aquatic products; vegetables; fruits; dried fruits; snacks; condiments and salt. The iodine content of foods was measured, considering the contribution of each food to the iodine content. The average daily dietary iodine intake of pregnant and lactating women was estimated using a FFQ to determine the frequency and consumption of the above eleven food groups during the last 3 months. The quantitative standards for food intake refer to the Retrospective Dietary Survey Supplementary Reference Food Atlas developed by Wang^(21)^. Dietary iodine intake was mainly derived from food, drinking water and iodised salt and was calculated as follows: dietary iodine intake = Σ (intake of each type of food × iodine concentration of each type of food + drinking water × water iodine concentration + salt intake × salt iodine concentration × (1–20 %), where 20 % is the cooking loss rate of iodised salt as defined by the WHO^(22)^. The water iodine content was derived from the data of survey results on water iodine content in Xinjiang in 2017 (2017 Xinjiang-wide water iodine monitoring data [unpublished data]). Salt iodine content was obtained from the data of iodine deficiency disease monitoring salt iodine in the Xinjiang region in 2021 (2021 Xinjiang-wide iodine deficiency disease surveillance data [unpublished data]).

The final section of the questionnaire assessed knowledge, attitude and behaviours about iodine. This questionnaire was designed based on a review of the literature from national and international resources. The content was verified by three experts before use, and the validity of the content of the questionnaire was confirmed. Participants were asked what they knew about the following questions: (1) Who is the key population that needs iodine supplementation? (2) What are the most important dietary sources of iodine? (3) Do you think you live in an iodine-deficient area? (4) What do you know about the current iodine status in Xinjiang? Correct answers were assigned 2 points, correctly identified false answers were assigned 1 point and incorrect answers received 0 points. According to the knowledge score of each knowledge question, the total knowledge score was assessed ranging from 0 to 23, which was divided into low knowledge (0–7 points), medium knowledge (8–15 points) and high knowledge (16–23 points). Participants were asked to evaluate attitudes and behaviours (see Fig. 1 for the specific questions). Some questions had multiple answers to choose from and more than one correct answer, such as questions 1, 2, 8, 9, 10 and 11.

### Urine sample collection and laboratory measurements

Spot urine samples were collected to determine the UIC among pregnant and lactating women. Urine cups and numbered microcentrifuge tubes (EP tubes) were given to participants, who were told to use the urine cups to obtain 5 ml of a midstream urine and pour the collected urine into numbered EP tubes. The EP tubes were then given to the site investigator who was responsible for collecting the urine specimens. Oral instructions about urine collection and disposal were given to the pregnant and lactating women. To ensure the accuracy of urinary iodine determination, all urine samples were stored at –20°C and transported to the Laboratory of the Environmental Health and Endemic Disease Control Division of the Xinjiang Uygur Autonomous Region Center for Disease Control and Prevention through a cold chain system, where the UIC was determined. The UIC was determined according to the Arsenic-Cerium Catalytic Spectrophotometric Determination of Iodine in Urine protocol (WS/T 107-2016)^(23)^. The main instruments and reagents were as follows: UV–Vis spectrophotometer (UNICO UV-4802; Shanghai Unico Instrument Co., Ltd.); and arsenic-cerium catalytic spectrophotometry kit (lot number 04 202 085; Wuhan Zhongsheng Biochemical Technology Co., Ltd.).

### Definitions of iodine status and recommendations for iodine intake

In the current study, the MUIC recommended by the WHO was used to evaluate the iodine nutritional status of the population. Specifically, an MUIC < 150 µg/l and < 100 µg/l are considered inadequate iodine nutritional status in pregnant and lactating women, respectively^(4)^. In the 2017 Chinese Residents’ Reference, Dietary Intake of Nutrients (Part 3: Trace Elements), the recommended nutrient intake (RNI) for pregnant and lactating women are 230 µg/d and 240 µg/d, respectively, and the tolerable upper intake level is 600 µg/d^(24)^.

### Statistical methods

Data were analysed using IBM SPSS statistics (version 26). The Kolmogorov–Smirnov test was used to determine whether outcome variables were normally distributed. The results are expressed as the mean (sd) for parametric continuous data, the median and 25th–75th percentile (p25–p75) for non-parametric continuous data and frequencies for categorical data. Comparisons between groups were carried out using a *x^2^* test for categorical data, a *t* test or one-way ANOVA for continuous parametric data, the Mann–Whitney U test for continuous non-parametric data and Spearman correlation to examine agreement between continuous variables. The UIC and dietary iodine intake were not normally distributed, while iodine knowledge scores were normally distributed. Participants were divided into three groups (inadequate, appropriate or excessive dietary iodine intake). Insufficient and appropriate intake, excess and appropriate intake were used as dependent variables, respectively, and variables with a *P* < 0·05 in the univariate analysis were included as the independent variables for binary logistic regression analysis. The forward LR partial likelihood estimation method was used in the regression analysis. For all data, a *P*-value < 0·05 was considered statistically significant.

## Results

### Basic characteristics of pregnant and lactating women

Table 1 outlines the demographic characteristics of the participant. A total of 1701 women participated in this study; 1181 pregnant and 504 lactating women completed the questionnaire regarding iodine nutritional knowledge (response rate = 99·06 %). The mean age of pregnant and lactating women was 26·78 years (sd = 4·89 years) and 27·05 years (sd = 4·91 years), respectively. The average gestational age (in weeks) of pregnant women was 23·51 weeks (sd = 1·12 weeks), mostly in the second trimester, followed by the third trimester. In addition, this was the first successful pregnancy for 48·8 % of the pregnant women and the first birth for 54·4 % of the lactating women. Meanwhile, all participants consumed iodised salt, with 100 % coverage of iodised salt.

**Table 1:** Basic characteristics of pregnant and lactating women*

	Pregnant women (*n* 1181)		Lactating women (*n* 504)	
Characteristics	*n*	%	*n*	%
Age (years)				
Mean	26·78		27·05	
sd	4·89		4·91	
< 25 years	554	47·0	224	44·4
25–35 years	558	47·2	252	50·0
> 35 years	69	5·8	28	5·6
Weight (kg)				
Mean	57·28		57·71	
sd	9·93		5·62	
Height (cm)				
Mean	161·36		161·12	
sd	7·21		5·62	
BMI (kg/m^2^)†				
Mean	22·02		22·24	
sd	3·64		3·36	
Underweight	181	15·3	53	10·5
Normal	701	59·4	320	63·5
Overweight	229	19·4	101	20·0
Obesity	70	5·9	30	6·0
Education classification‡				
Primary and below	115	9·7	32	6·3
Middle school education	511	43·3	263	52·2
High school and junior college	332	28·1	122	24·2
College education or above	223	18·9	87	17·3
Annual household income (CNY)§				
< 20 000	159	13·5	83	16·5
20 000–50 000	752	63·7	312	61·9
50 000–100 000	219	18·5	77	15·3
≥ 100 000	51	4·3	32	6·3
Employment||				
Yes	544	46·1	290	57·5
No	637	53·9	214	42·5
Gestational weeks				
Mean	23·51		–	
sd	1·12		–	
First trimester	187	15·8	–	
Second trimester	537	45·5	–	
Third trimester	457	38·7	–	
Parity				
Primiparity	576	48·8	274	54·4
Multiparity	604	51·2	230	45·6
Passive smoking				
Yes	780	66·0	185	36·7
No	401	34·0	319	63·3
History of abortion				
Yes	294	24·9	113	22·4
No	887	75·1	391	77·6
Thyroid nodules				
Yes	164	13·9	97	19·2
No	1017	86·1	407	80·8
Received education about iodine¶				
Yes	643	54·4	327	64·9
No	380	32·2	109	21·6
Do not remember	158	13·4	68	13·5
Active iodine supplementation awareness**				
Yes	508	43·0	226	44·9
No	417	35·3	165	32·7
Not sure	256	21·7	113	22·4

* Values are presented as the Mean (sd), Median (IOR), or *n* (%).

† For pregnant women, BMI was calculated from the pre-pregnancy weight. BMI < 18·5 kg/m^2^: underweight; 18·5 ≤ BMI < 24·0 kg/m^2^: normal; 24·0 ≤ BMI < 28·0 kg/m^2^: overweight; BMI ≥ 28·0 kg/m^2^: obesity.

‡ Tertiary education is college education or above; others are non-tertiary education.

§ CNY is Chinese Yuan.

|| Defining housewives and farmers as non-employed.

¶and **are questions 5 and 6, respectively.

### Iodine knowledge scores and iodine knowledge question responses

The average total score of iodine knowledge for pregnant and lactating women was 12·59 (sd = 2·66) and 12·27 (sd = 2·86), respectively, without a maximum score of 22 and no scores of 0 (Table 2). Pregnant women had slightly higher iodine knowledge scores than lactating women (*P* < 0·05). The majority of participants had a medium level of iodine knowledge (73·4 % of pregnant women and 82·5 % of lactating women). Furthermore, for pregnant and lactating women, no differences existed in iodine knowledge scores according to education, employment, trimester and parity (all *P* > 0·05); however, the iodine intake of pregnant women was positively associated with iodine knowledge scores (*r* = 0·089, *P* < 0·05). On further analysis of the data, although pregnant and lactating women who had received iodine education had higher iodine knowledge scores than those who did not, the difference was not statistically significant (all *P* > 0·05; Table 3).

**Table 2:** Iodine knowledge scores among pregnant and lactating women*

	Pregnant women (*n* 1181)		Lactating women (*n* 504)	
Iodine knowledge score	*n*	%	*n*	%
Total score†				
Mean	12·59		12·27	
sd	2·66		2·86	
Low knowledge	152	12·9	20	4·0
Medium knowledge	867	73·4	416	82·5
High knowledge	162	13·7	68	13·5

* Values are presented as the Mean (sd), or *n* (%). Differences in the score were tested with independent samples *t* test and one-way ANOVA.

† Scores range from 0 to 23.

**Table 3:** Urinary iodine concentration (UIC), dietary iodine intake, food iodine intake only and iodine knowledge scores among pregnant and lactating women with iodine education and higher education. (Medians and interquartile ranges (IQR)

	Pregnant women										Lactating women									
	All women (*n* 1181)		No received iodine education (*n* 111)		Received iodine education (*n* 841)		Non-tertiary education (*n* 957)		Tertiary education (*n* 223)		All women (*n* 504)		No received iodine education (*n* 39)		Received iodine education (*n* 384)		Non-tertiary education (*n* 417)		Tertiary education (*n* 87)	
	M‡	IQR	M‡	IQR	M‡	IQR	M‡	IQR	M‡	IQR	M‡	IQR	M‡	IQR	M‡	IQR	M‡	IQR	M‡	IQR
Food iodine intake only (µg/d)	97·21	66·85, 163·90	129·14	74·73, 198·90	95·32*	67·33, 150·14	94·70	64·39, 150·07	129·78*	81·58, 194·07	91·52	55·78, 169·29	135·46	88·83, 185·60	80·03*	54·42, 158·85	84·25	54·21, 155·38	147·92*	65·76, 225·18
Dietary iodine intake (µg/d)	407·16	320·40, 513·21	454·95	345·45, 541·49	398·51*	319·76, 504·49	394·21	317·09, 504·37	441·24*	352·04, 537·26	356·89	304·33, 485·63	452·38	370·91, 594·97	344·45*	302·74, 458·42	347·51	302·43, 453·29	483·39*	343·95, 563·23
UIC (µg/l)	179·27	106·83, 260·60	174·34	126·91, 265·81	177·76	106·33, 258·68	176·85	104·56, 260·60	195·90	119·13, 256·80	192·81	113·35, 276·77	142·20	76·64, 210·13	198·74	119·17, 287·05	198·74	119·23, 289·31	153·70*	90·82, 215·93
Iodine knowledge scores†																				
Mean	12·60		12·57		12·60		12·55		12·72		12·27		12·44		12·49		12·23		12·47	
sd	2·66		2·71		2·67		2·64		2·73		2·86		2·82		2·83		2·87		2·85	

There were three answers available to the question of whether they had received iodine nutrition education during pregnancy: they had, they had not and not sure if they had. Pregnant and lactating women who were unsure whether they had received education on iodine nutrition were 229 and 81 (data not shown), respectively. Therefore, the food iodine intake only, the dietary iodine intake, UIC and iodine knowledge scores were first compared between these three groups, with differences followed by multiple comparisons.

* Statistically significant difference compared to non-tertiary education and no received iodine education (*P* < 0·05).

† Mean (sd).

‡ Median.

**Fig. 2:**
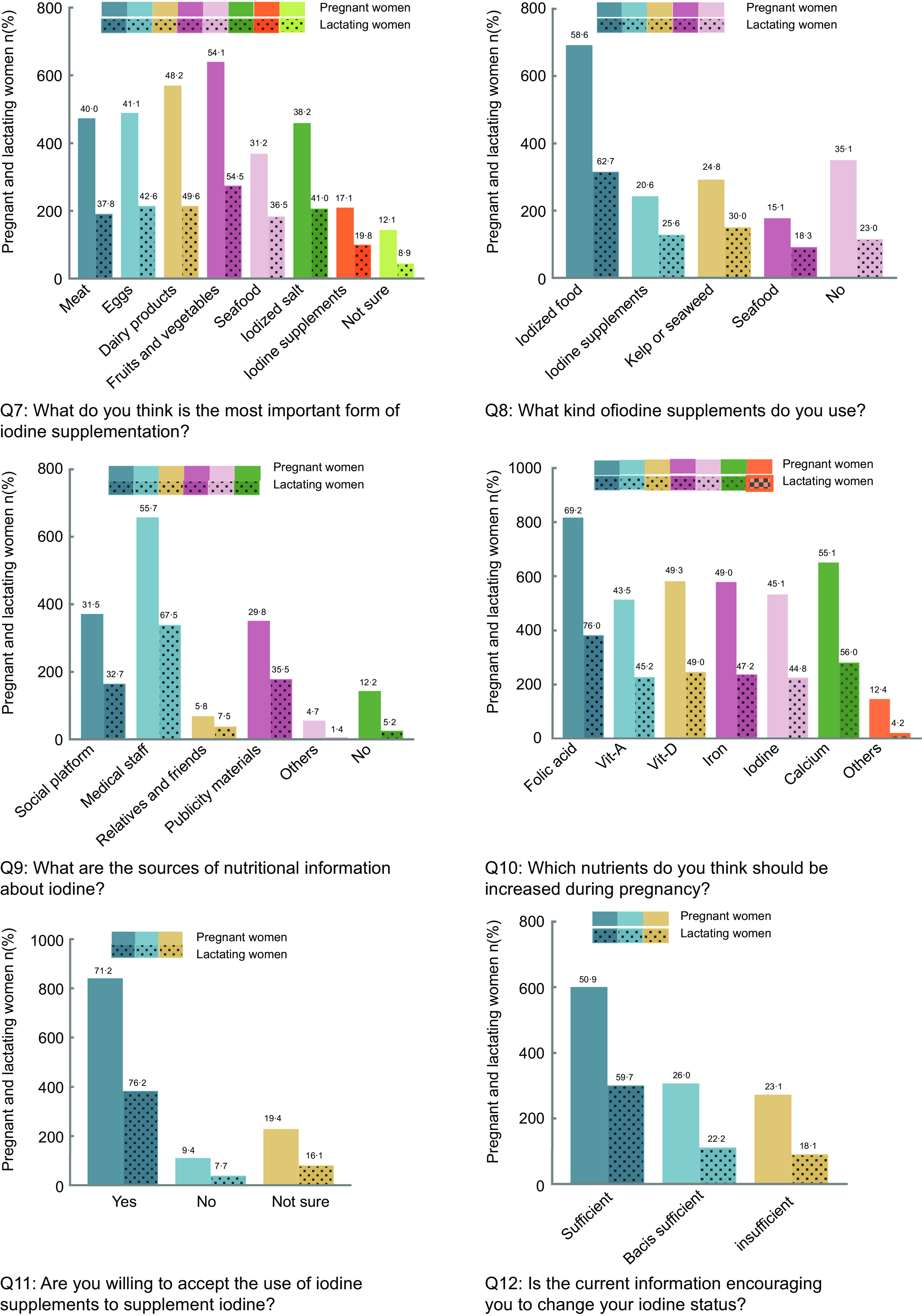
Information on attitudes, behaviours about iodine nutrition, and sources of iodine knowledge for pregnant and lactating women

Data regarding iodine knowledge applied to determine knowledge scores are summarised in Table 4. Pregnant and lactating women were correctly recognised as population groups with higher iodine requirements by 73·6 % and 47·5 % of pregnant women and 69·4 % and 61·9 % of lactating women, respectively, but approximately one-third of pregnant and lactating women each mistakenly believed that ordinary adults were the population with higher iodine requirements. Nearly one-half of pregnant and lactating women correctly identified eggs (45·0 % and 49·0 %, respectively) and dairy products (48·8 % and 50·2 %, respectively) as the most important dietary iodine sources, followed by seafood (37·1 % and 41·1 %, respectively). Vegetables, meat and fruits were mistakenly considered iodine-rich foods by some pregnant and lactating women (see Fig. 2 for specific data), but only 6 % of pregnant and 3 % of lactating women indicated they did not know what listed foods were good sources of iodine. Pregnant women with a tertiary education had significantly better knowledge of the dietary sources of iodine than pregnant women without a tertiary education (*P* < 0·001 (data not shown)). In addition, the Xinjiang region is an iodine-deficient region correctly identified by only 20·2 % of pregnant women and 26·8 % of lactating women. The current nutrient status in Xinjiang was considered appropriate by 39·7 % of pregnant women and 48·2 % of lactating women, whereas around 30 % of pregnant and lactating women were unsure.

**Table 4:** Summary of questions about iodine knowledge for determining iodine knowledge scores for pregnant and lactating women *

	Pregnant women (*n* 1181)		Lactating women (*n* 504)	
Iodine knowledge	*n*	%	*n*	%
Key population with higher iodine supplementation†				
Infant	444	37·6	210	41·7
Children	591	50·0	252	50·0
Pregnant women‡	869	73·6	350	69·4
Lactating women‡	561	47·5	312	61·9
Adults	393	33·3	174	34·5
Do not know	124	10·5	33	6·5
Most important dietary iodine sources†				
Eggs‡	532	45·0	247	49·0
Milk products‡	576	48·8	253	50·2
Meat products	512	34·4	245	48·6
Vegetables	550	46·6	293	58·1
Fruits	424	35·9	199	39·5
Seafood‡	438	37·1	207	41·1
Cereals	117	9·9	38	7·5
Pasta	168	14·2	62	12·3
Other food	77	6·5	16	3·2
Do not know	71	6·0	16	3·0
Whether you live in an iodine-deficient area				
Yes‡	238	20·2	135	26·8
No	561	45·7	226	44·8
Do not know	382	32·3	143	28·4
The current iodine status in Xinjiang				
Iodine deficiency	270	22·9	113	22·4
Iodine appropriateness‡	469	39·7	243	48·2
Iodine super suitable	42	3·6	19	3·8
Iodine excess	11	0·9	2	0·4
Do not know	389	32·9	127	25·2

* Values are presented as *n* (%).

† Multiple answers are possible.

‡ Correct answer.

### Attitudes, behaviours and sources of information about iodine nutrition

Figure 2 summarises information on attitudes, behaviours and sources of knowledge regarding iodine nutrition. The main sources of information related to iodine nutrition for pregnant and lactating women were the medical and nursing staff (55·7 % and 67·5 %, respectively), followed by social platforms (31·5 % and 32·7 %, respectively), as well as publicity materials (29·8 % and 35·5 %, respectively), whereas 12·2 % of pregnant and 5·2 % of lactating women could not remember whether or not they had received this information. Significant differences in the sources of information received by pregnant and lactating women were associated with age (*P* < 0·05) and education (*P* < 0·05). The nutritional information currently available was sufficient to satisfy nutritional status according to 50·9 % of pregnant women and 59·7 % of lactating women.

The information provided about specific nutrients (folic acid, Fe, iodine, Ca and vitamins A and D) varied. Compared with other nutrients, iodine awareness among pregnant and lactating women remained inadequate, with 45·1 % of pregnant women and 44·8 % of lactating women believing they needed to increase iodine intake during pregnancy, while less than half (43·0 % of pregnant women and 44·8 % of lactating women) reported taking action to intentionally change their dietary habits to increase iodine intake during pregnancy. Furthermore, pregnant women with a tertiary education were more likely to be proactive in iodine supplementation during pregnancy (36·9 % *v*. 6·1 %, *P* < 0·05 (data not shown)), whereas there was no difference in the lactating women (*P* > 0·05). Approximately two-thirds of the participants selected iodine-enriched foods to supplement their iodine nutrition (58·6 % of pregnant women and 62·7 % of lactating women), followed by seaweed or kelp and iodine supplements (see Fig. 2 for specific data). Only 38·9 % of pregnant women and 41·1 % of lactating women believed that the most important form of iodine supplement was iodised salt.

A statistically significant difference (54·4 % of pregnant and 64·9 % of lactating women) reported that they had been educated about iodine, and compared with women who had not been so educated, these women would be more likely to increase iodine intake due to pregnancy (*P* < 0·001) and more willing to accept the use of iodine supplements for iodine nutrition (*P* < 0·001 [data not shown]).

### Urinary iodine concentration and iodine status

**Fig. 3:**
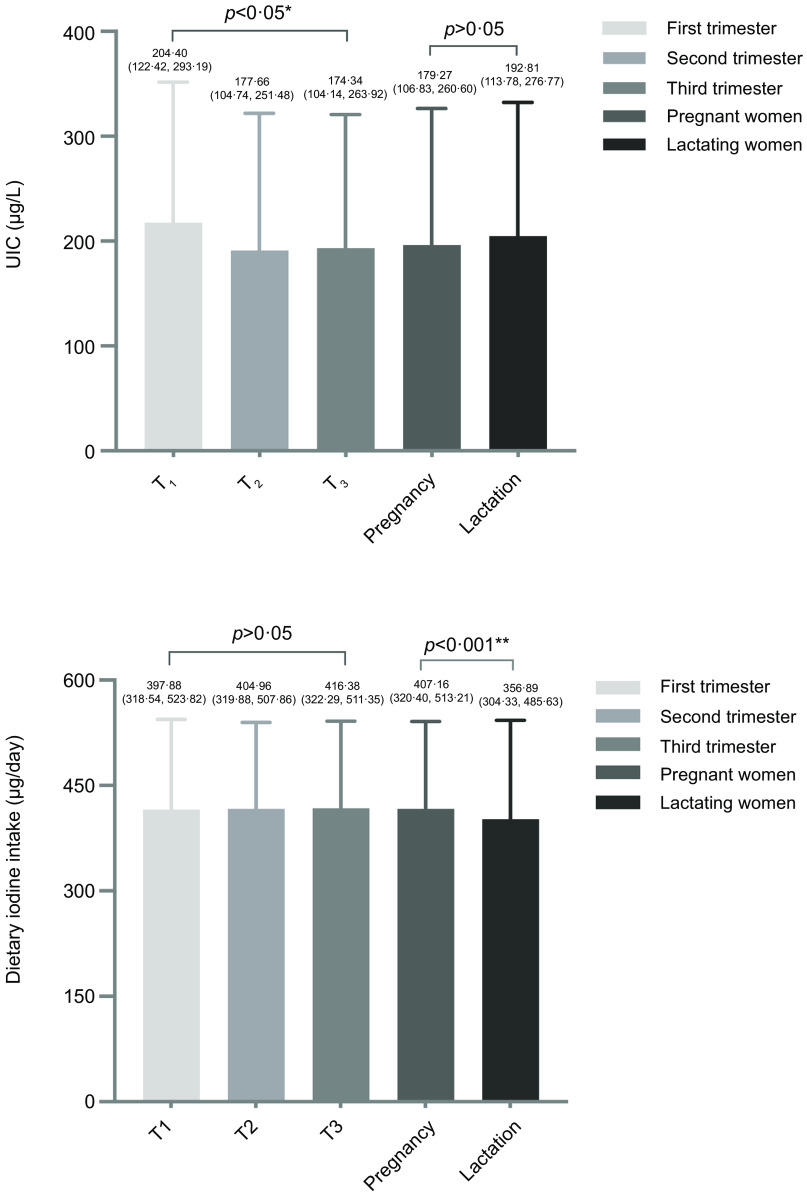
Urinary iodine concentration (UIC) and dietary iodine intake for pregnant and lactating women. *Significant (*P* < 0·05); **Significant (*P* < 0·001); T1 is first trimester; T2 is second trimester; T3 is third trimester

For all participants, the MUIC of pregnant women was 179·27 µg/l with an IOR of 106·83–260·60 µg/l and the MUIC of lactating women was 192·81 µg/l with an IOR of 113·78–276·77 µg/l. According to the WHO criteria, the MUIC was inadequate in 38·2 % of pregnant women and 20·2 % of lactating women. A comparison of the UIC between women in the pregnant and lactating groups revealed no significant difference (*P* > 0·05). There was, however, a significant difference in the UIC among the gestational age groups (204·40 µg/l, 177·66 µg/l and 174·34 µg/l for women in the first, second and third trimesters, respectively, *P* < 0·05; Fig. 3).

There was no association between the UIC and general demographic characteristics in pregnant women (all *P* > 0·05), whereas the UIC in lactating women was significantly different based on the age group (*P* < 0·05) and education level (*P* < 0·05). UIC in pregnant and lactating women did not differ between groups with iodine education, without iodine education and unsure if they had iodine education (*P* < 0·05); UIC was lower among lactating women with tertiary education than non-tertiary education, with statistically significant difference (*P* < 0·05), but there was no difference between tertiary and non-tertiary education groups for pregnant women (*P* > 0·05; Table 3). The correlation analysis of the data showed a positive correlation between the UIC and dietary iodine intake (*r* = 0·064, *P* < 0·05) in pregnant women, and a negative correlation between the UIC and age (*r =* –0·118, *P* < 0·05), number of births (*r =* –0·093, *P* < 0·05) and dietary iodine intake (*r =* –0·102, *P* < 0·05) in lactating women (Table 5 (data on UIC Spearman correlation analysis not shown)).

**Table 5:** Correlation analysis between dietary iodine intake and selected human indices and UIC and iodine knowledge in pregnant and lactating women†

	Pregnant women (*n* 1181)		Lactating women (*n* 504)	
Variables	*r*	*P*-value	*r*	*P*-value
Age (years)	0·160	< 0·001**	0·120	0·007*
Weight (kg)	0·056	0·053	–0·139	0·002*
Height (cm)	0·084	0·004*	0·044	0·321
BMI (kg/m^2^)‡	0·024	0·406	–0·156	< 0·001**
Gestational weeks	0·039	0·188	–	–
Number of births	0·081	0·005*	0·039	0·387
UIC (µg/l)§	0·064	0·028*	–0·102	0·022*
Iodine knowledge scores||	0·089	0·002*	0·002	0·961

* Significant (*P* < 0·05).

** Significant (*P* < 0·001).

† Spearman correlation was used to analyse the relationships between dietary iodine intake and age, weight, height, BMI, gestational age in weeks, number of births, UIC and iodine knowledge scores.

‡ For pregnant women, BMI is calculated from pre-pregnancy weight.

§ UIC, urinary iodine concentration (µg/l).

|| Scores range from 0 to 23.

### Relationship between iodine knowledge and dietary iodine intake

Complete dietary iodine intake data were obtained from all participants. The median (IQR) iodine intake for pregnant and lactating women was estimated as 407·16 µg/d (range, 320·40–513·21 µg/d) and 356·89 µg/d (range, 304·33–485·63 µg/d), respectively, which met the daily iodine recommendation (RNI of 230 µg/d and 240 µg/d for pregnant and lactating women, respectively). For the vast majority of participants (88·8 % of pregnant women and 87·3 % of lactating women), the average daily dietary iodine intake was appropriate. Dietary iodine intake differed between pregnant and lactating women and was higher in pregnant women than in lactating women (*P* < 0·001; Fig. 3). In addition, the dietary iodine intake of pregnant and lactating women in the Yili region of northern Xinjiang was significantly higher than the Aksu region of southern Xinjiang (*P* < 0·05 [data not shown]).

In addition to the knowledge scores, dietary iodine intake was positively associated with age (*r* = 0·160, *P* < 0·001), height (*r* = 0·084, *P* < 0·05) and number of births (*r* = 0·081, *P* < 0·05) for pregnant women, but only positively correlated with age (*r* = 0·120, *P* < 0·05; Table 5) for lactating women. Moreover, pregnant and lactating women who received iodine education and were willing to receive iodine supplements were likely to have appropriate iodine intake status (all *P* < 0·05 [data not shown]). Those participants who changed their dietary habits to increase iodine intake did not have a higher iodine intake than other pregnant and lactating women, although the difference was statistically significant (*P* < 0·05 [data not shown]). Pregnant and lactating women with tertiary education had higher food iodine intake and higher total dietary iodine intake than pregnant and lactating women with non-tertiary education (*P* < 0·05; Table 3).

### Other factors affecting iodine intake in pregnancy and lactation

**Fig. 4:**
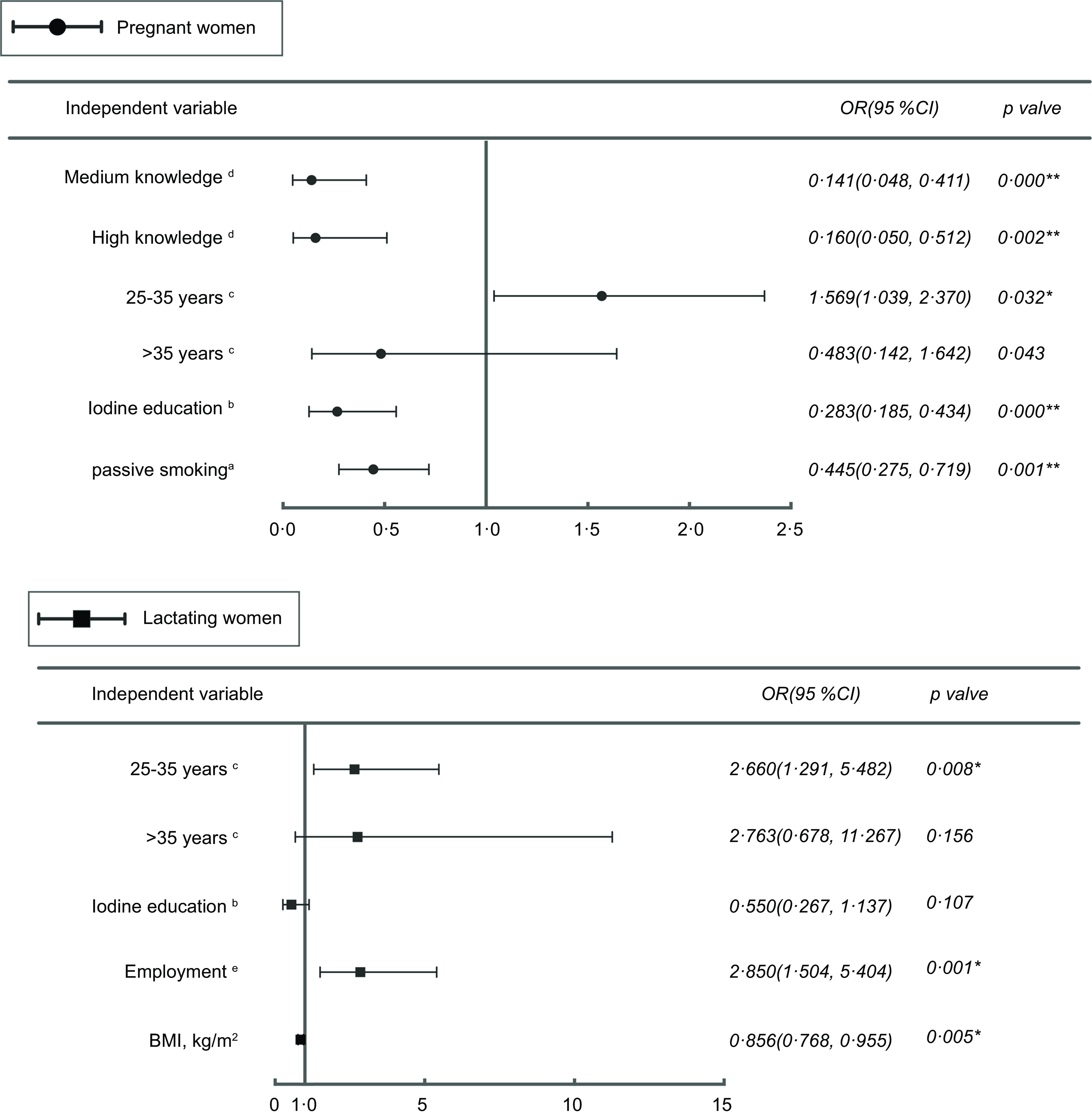
The factors associated with dietary iodine intake. ^a, b, c, d, e^ for no-passive smoking, no iodine education, < 25 years, low knowledge scores, and no employment were the references, respectively. Dependent variable: iodine intake (appropriate and excess). *Significant (*P* < 0·05); ** Significant (*P* < 0·001)

The results of the analysis of factors affecting dietary iodine intake are shown in Fig. 4. The results of the binary logistic regression analysis of excess iodine intake in pregnant women showed four variables in the model: (i) categorical age variable; (ii) passive smoking; (iii) receipt of iodine education and (iv) iodine knowledge scores. High scores for iodine knowledge *v*. low scores (*P* < 0·05), receipt of education about iodine *v*. no receipt of education about iodine (*P* < 0·001) and passive smoking *v*. no passive smoking (*P* < 0·05) may thus serve as protective factors for excess iodine intake in pregnant women. The results of the binary logistic regression of excess iodine intake in lactating women showed four variables in the model: (i) BMI; (ii) age categorical variable; (iii) employment and (iv) receipt of iodine education. Compared to pregnant and lactating women < 25 years of age, pregnant and lactating women 25–35 years of age (*P* < 0·05), and employment for lactating women *v*. no employment (*P* < 0·05), were shown to be risk factors for excess dietary iodine intake. For each incremental increase in the BMI, the risk of excess iodine intake in pregnant women decreased by 0·856-fold (*P* < 0·05). All of the included independent variables that may have affected inadequate iodine intake in pregnant women had *P* values > 0·05. The only independent variables that may have affected inadequate iodine intake in lactating women were employed, with no employment as the reference (OR, 4·547; 95 % CI, 1·541, 13·411; *P* < 0·05 [data on inadequate iodine intake not shown]).

## Discussion

This cross-sectional study presented the current status of iodine nutrition and knowledge among pregnant and lactating women in Xinjiang. A majority of women were shown to have appropriate dietary iodine intake and a medium level of iodine knowledge.

According to the lower limit of appropriate iodine nutritional status for pregnant and lactating women (MUIC < 150 µg /l and 100 µg /l, respectively), as recommended by the WHO, women in our study were in an appropriate iodine nutritional status, and the results about pregnant women were similar to those reported in a previous surveillance study conducted in Xinjiang^(18,25)^. There were differences in the MUIC among pregnant women in different trimesters, with significantly higher MUIC in early than late pregnancy, which is consistent with the results of the Shanghai^(26)^, Chongqing^(27)^ and Australian studies^(28)^. This finding may be related to the increased demand for TH by the foetus. During the first 20 weeks of pregnancy, the foetus is completely dependent on the maternal supply of TH. When the foetal thyroid gland matures, maternal iodine is transferred to the foetus, increasing the maternal demand for iodine^(3,26)^. Pregnant women in late pregnancy are advised to increase the intake of iodine-rich foods. Increased iodine intake in lactating women was associated with decreased UIC. Dold et al.^(29)^ suggested that maternal UIC alone may not reflect the iodine status of lactating women. In lactating women with adequate overall iodine status, iodine excretion in breast milk may be increased and the urinary iodine excretion fraction may be decreased^(29)^.

Adequate dietary iodine intake is essential for the production of TH, and TH are critical for the normal growth and neurodevelopment of pregnant and lactating women and foetuses. Compared to non-pregnancy, pregnant and lactating women have a ≥ 50 % increase in iodine requirements^(30)^.

Our study showed that pregnant and lactating women consumed less iodine from food alone than the RNI for non-pregnant women (150 μg/d), but total dietary iodine intake (including food, salt and drinking water) met the lower limit of the WHO RNI for pregnant and lactating women (250 μg/d) and met the RNI requirements in China; specifically, the daily iodine intake for pregnant and lactating women should be at least 230 μg and 240 μg, respectively^(31)^. This finding can be attributed to universal salt iodisation, which aims to ensure adequate iodine intake for all people. In 2014, the results of China’s IDD survey suggested that > 90 % of households consume adequately iodised salt^(14)^. The relevant survey showed that the coverage rate of iodised salt in Xinjiang also reached > 90 % and achieved a qualified rate, which was consistent with the results of our study^(18,25)^. Iodised salt has been shown to predict iodine intake levels^(17,30)^. Xinjiang has made good progress in eliminating IDD due to the implementation of universal salt iodisation^(18)^; however, iodised salt was correctly identified as an important iodine supplement by only one-third of the women in this study. This finding indicated that the importance of iodised salt should be strongly promoted, and misconceptions of the public about iodised salt should be addressed. Meanwhile, this study focussed on special women’s intake of iodine-rich foods mainly because we found through the literature review that even in some areas where salt iodisation programs have been established, pregnant women may have inadequate iodine intake^(32–34)^. Although salt iodisation is the simplest and most effective form of iodine supplementation, excessive salt intake can increase Na levels in the body because of the high iodine requirements of pregnant and lactating women, increasing the risk of gestational hypertension, osteoporosis and Ca deficiency^(35–37)^. The Dietary Guidelines for Chinese Residents released in 2022 recommend that Chinese residents over eleven should not consume more than 5 g of salt per day and that pregnant women maintain a light, palatable, low-salt diet^(38)^. Therefore, it is also essential to pay attention to the intake of iodine-rich foods by special women under the national salt iodisation measure.

The majority of pregnant and lactating women in this study had medium levels of iodine knowledge. Iodine knowledge deficiency has been demonstrated in both populations in some countries^(11,13,39,40)^. We found that pregnant women had higher iodine knowledge scores than lactating women, which contrasts with the results of a Norwegian study^(13)^. In addition, Charlton et al.^(40)^ did not find differences in iodine perceptions between pregnant and lactating women. Contrary to expectations, UIC was not shown to be associated with iodine knowledge scores in pregnant and lactating women, which is consistent with the results of a Norwegian study^(13)^. In a study of pregnant women in Shanghai^(26)^ and a study of 2642 pregnant women in Zhejiang Province, China^(17)^, the MUIC was shown to increase significantly with increasing iodine knowledge scores. There was no variability in iodine knowledge scores among other personal characteristics among pregnant and lactating women. Several studies have indicated that different cultural, environmental and interpersonal factors may have negative or positive effects on nutritional knowledge levels of individuals^(41,42)^.

In the present study, there was a positive correlation between iodine knowledge and dietary iodine intake among pregnant women. This finding was consistent with results from the UK and Ireland and Norway in women of childbearing age^(43,44)^. Increased iodine knowledge may be important to ensure adequate iodine intake. The relatively small sample size of lactating women in the current study may explain the lack of correlation between iodine knowledge and iodine intake; however, there was confusion among women in identifying the main food sources of iodine. Similarly, other existing studies have shown a lack of knowledge about the main dietary sources of iodine in pregnant and lactating women^(6,13)^. The Dietary Guidelines for Chinese Residents recommend that pregnant women should consume seafood at least one time per week, but less than one-fifth of women take iodine supplements through seafood consumption^(38)^. This phenomenon was not only due to a lack of awareness about iodine-rich foods among women but was also related to the geographic environment of Xinjiang, which is far from the sea and deep inland. External environmental iodine deficiency and current appropriate iodine nutritional status in Xinjiang were only correctly identified by a few women. Therefore, information and education about iodine are essential to raise public awareness of nutritional health.

May 15 is designated as the National Iodine Deficiency Disorders Prevention Day in China; on which day the government and hospitals conduct education and awareness activities on iodine nutrition. In a cross-sectional survey of 1026 women in the UK, 87 % reported that they would be willing to change their dietary behaviour to increase their iodine intake if they received information related to the importance of iodine during pregnancy^(6)^. This finding was consistent with our finding that women who had received iodine education and information exhibited proactive behaviours and attitudes towards iodine nutrition. An Iranian educational intervention with pregnant women for 4 months also increased their iodine knowledge, attitudes and behaviours^(42)^. Other studies have confirmed that iodine education is one of the predictors of iodine knowledge scores^(45)^. In the present study, iodine education is a protective factor for appropriate iodine intake in pregnant women.

Medical staff were selected as the primary source of information on iodine knowledge, which was consistent with other studies involving pregnant or lactating women^(39,40)^, whereas studies in the UK and Australia concluded that some healthcare professionals have low knowledge of iodine, its sources or its role in health^(46,47)^. Therefore, it is necessary to improve the knowledge of medical staff about iodine and other nutrients, especially for some poor and low education level areas. In addition, more women also gained iodine knowledge through online social platforms. Short message service intervention has been shown to improve the knowledge and attitudes of women regarding iodine deficiency and iodised salt consumption in Tehran^(48)^. In addition to the traditional distribution of pamphlets on iodine knowledge, local governments and other authorities can also use social platforms, such as WeChat and Weibo, to improve attitudes towards active iodine supplementation.

The growth and development of infants and children require the support of multiple micronutrients. Growth and cognitive deficits in children caused by malnutrition may hinder learning and skill development. Maternal education and knowledge of nutrition have a significant impact on the survival, growth and development of infants and children^(49)^. As Block^(50)^ suggested, the content and quality of micronutrients in foods are correctly identified by more knowledgeable and educated consumers, and the mother’s education may be an important factor in determining the nutritional status of children. Moreover, we found that years of education were associated with iodine intake in pregnant and lactating women, perhaps because these mothers made more careful and focussed choices about dietary nutrient intake. After multifactorial analysis, years of education were no longer an influential factor in women’s inadequate and excessive iodine intake; however, pregnant women who are educated in iodine nutrition and have a high iodine knowledge may have a positive effect on controlling excessive iodine intake. Based on the level of education and different level of nutrient knowledge among pregnant and lactating women in Xinjiang, targeted public campaigns are conducted to improve their different nutritional knowledge and awareness of active iodine supplementation, thus contributing to the healthy growth and development of infants and children. In addition, it is also significant to address gaps in public knowledge regarding most dietary sources of iodine and misconceptions about iodised salt to prevent some people from consuming less than the RNI or more than the upper intake level.

These findings indicate that although good progress has been made in eliminating IDD in the Xinjiang region of China, there may be deficiencies in knowledge dissemination and education about iodine and other nutrients. The limitation of this study was that the results were not representative of pregnant and lactating women in places other than the Xinjiang region. In addition, while the UIC is a good indicator to evaluate the iodine intake of the population, a single random UIC cannot be used to represent the iodine nutritional status of an individual due to the susceptibility of drinking water and food. Also, the breast milk iodine concentration is a more accurate biomarker of iodine status than the UIC in lactating women^(29,51)^. Self-report bias in participants completing the questionnaire could not be excluded from this study. The strengths of this study include the relatively large sample size of participants, who were pregnant and lactating women from two different geographic regions in the Xinjiang region. In addition to data related to iodine knowledge, data on dietary iodine intake and urinary iodine were also available. We collected and tested the iodine content of the different types of foods covered by the FFQ. This study has filled the gaps in data from studies related to iodine knowledge levels of pregnant and lactating women in Xinjiang.

## Conclusion

In the present study, the iodine status and dietary iodine intake of pregnant and lactating women were appropriate, but the women had only a medium iodine knowledge. In addition, we discovered that the women lacked knowledge about important dietary sources of iodine. We found that the iodine knowledge scores of pregnant women were positively correlated with dietary iodine intake, and that having high iodine knowledge scores and having received iodine education may be influential factors that prevent pregnant women from consuming too much iodine.
